# Quarterly Summary

**Published:** 1858-01

**Authors:** 


### QUARTERLY SUMMARY.

1.—*Registers of the Human Voice.*—We find in the September number of the Peninsular Journal of Medicine, an exceedingly interesting article on this obscure and difficult subject.

The author, Dr. E. Andrews, of Chicago, sets out by defining the three registers, commonly known as the *chest*, *medium* and *head* voices or registers. He calls attention to the fact that they do not differ merely in *pitch*, since there are certain notes which can be given in either register. He then calls attention to the anatomy of the larynx, especially the form and attachments of the arytænoid cartilages, their anterior and posterior processes, to the former of which the vocal ligaments are attached, and the fact that these must necessarily partake of the movements of that process. He then proceeds to question the commonly received opinion that the vocal chords are like the reed in the clarionet, and insists, on the contrary, that their vibration resembles that of the lips in blowing a sax horn or any of the trumpet instruments.

He strengthens his opinion by citing certain facts he observed in the larynx of a man who had cut his throat, completely shaving off the top of his larynx, just above the vocal ligaments. When the patient was silent, the arytenoid cartilages lay with their superior processes pointing upwards into the pharynx, and their anterior processes rotated outwards, so as to leave a wide opening between them, their points resting on the sides of the cavity of the larynx. When he spoke, however, all was changed. The anterior processes of the arytenoid swung inwards, describing one-eighth of a circle, till they came in contact, bringing the vocal ligaments together and stretching them tightly over the opening like a diaphragm, when the column of air from the chest passing over them produced a visible vibration in them.

The peculiar, reedy, strongly vibrating voice, called the chest register, is produced, according to our author, by bringing the edges of the vocal cord into pretty firm contact, so that considerable force is required in the column of air to open them and set them in vibration. In the larynx this close apposition is affected by the thyro-arytænoidei muscles, which by virtue of their attachments, rotate the anterior processes inward and so approximate the chords. Meanwhile the crico-thyroid muscles, by rotating the thyroid cartilage upon its inferior coruna, stretch the vocal chords, and by the different degrees of tension they thus produce, cause the formation of the different notes. These two sets of muscles antagonize each other, so that the effort to prolong the chest voice, especially becomes fatiguing, and the singer experiences a sensation of relief when he changes this for the medium register.

This register is produced, our author is persuaded, by partly relaxing the thyro-arytænoid muscles, so that the vocal chords shall not quite touch at their edges, but leave a narrow space between them. Hence less force is demanded in the column of air from the lungs, a fact of which every singer is conscious. The sense of relief is produced by the diminished tension of the thyro-arytenoid muscles, which enables the crico-thyroid to tighten the chords with less powerful contractions.

The head register of the female, which our author argues is the same with the falsetto of the male, he thinks is produced by a sort of stop arrangement in the larynx, which shortens the vocal ligaments, producing a higher tone, just as the shortening the string of a violin by pressing the finger on it gives it a higher note. He explains this by saying that when the cartilages are not firmly fixed, they must vibrate with the chords, thus forming practically part of the chords, and this he considers to be the condition of the parts during the production of the chest and middle registers. When the head voice, however, is used, according to his view the arytenoid cartilages are brought together and so firmly fixed by the muscles that they no longer vibrate as a part of the chords, but by their mutual pressure "stop" each other, and become fixed points like pegs to which the vocal ligaments are attached. The vibrating edges are thus shortened about one-third, so that the pitch of the sound is raised. Hence, the same amount of tension produces a much higher note than when the whole edge, arytenoid cartilages and all vibrated, so that the relief afforded to a female singer in passing into the head voice upon the high notes becomes intelligible. We would further suggest that perhaps the attempt of most female singers to carry their medium register up to the highest point, and their persisting in singing their upper notes as long as they can in that register, may afford an explanation of the commonly observed fact, that the note upon which the songstress changes to the head voice is apt to be the weakest in the whole range of her notes.

We have necessarily given but a brief abstract of this interesting paper. For the apposite illustrations from musical instruments by which the writer makes his meaning clear, we must refer our readers to the original publication.

---

2.—*Nervous Histology.*—A great advance in the histology of the nervous system has been recently made by Professor Jacubowitch.—From an abstract of his communication to the French Academies, copied from the *Comptes Rendus*, by the London Medical Times and Gazette, we make the following abridgement:

The cerebro-spinal system is made up of cells with axis-cylinders arising from their periphery. The cells are of three kinds: 1. *multipolar cells*, irregular in form, having many axis-cylinders passing off from their surface, usually 3 or 4; 2. *fusiform cells*, much smaller than the former, having usually 3 axis-cylinders, and 3. *bipolar cells*, oval in form, intermediate in size between the two other species, and having only two axis-cylinders. The *multipolar cells*, occur in the anterior cornua and gray substance of the spinal cord, in the cerebellum and in the corpora quadri gemina. They are not found in the medulla oblongata.

The *fusiform cells* occur in all the great centres. In the spinal cord they occupy the posterior cornua, and the cerebral hemispheres are almost entirely composed of them. The *bipolar cells* are absent from the hemispheres. In the spinal cord they are found chiefly about the central canal. The axis-cylinders of the *multipolar cells* form the nerves of motion, those of the *fusiform cells* the nerves of sensation, and those of the *bipolar cells* communicate with the great sympathetic. Hence the discoverer styles these three sets of cells respectively, cells of *motion*, cells of *sensation* and *ganglionary cells*. The cells of motion communicate with one another on the same side, and also with those on the opposite side through the anterior commissure. The posterior commissure does a similar service for the cells of sensation, while the axis-cylinders of the ganglionary cells pass indifferently by either commissure. The cells of motion predominate in the cervical and lumbar regions, those of sensation in the dorsal region of the spinal cord.

These elements are distributed among the centres as follows: The medulla oblongata is formed of cells of sensation and a nucleus of ganglionary cells. The cerebellum is made up of all these elements, the cells of motion reaching it by the pyramidal bodies, those of sensation by the restiform bodies, and the ganglionary cells passing in by both these lines of communication. Besides these, the cerebellum has a fourth order of cells, the pyriform cells peculiar to it, which send prolongations to the surface of the organ. The corpora quadrigemina also contain all these varieties, but the hemispheres have only the cells of sensation. Tho nerves of special sense contain only cells of sensation and ganglionary cells, but other nerves contain all these varieties.—Besides these elements Jacubowitch finds a special connective tissue binding these different cells together. In the different species of animals these cells vary in size, they are smallest in man, and in general are small in proportion to the intellectual development. The number of cells bears no proportion to the size of the brain.

3.—*Nerves of the Heart.*—Dr. Robert Lee, of London, who long ago acquired a high reputation by the demonstration of the nerves of the uterus, has recently followed in a still more brilliant discovery. He has succeeded in demonstrating a great quantity of nervous filaments and ganglia presiding over the movements of the heart. He has shown these to be present at all periods of life, and in all the mammalia he has as yet examined, and it increases in size in certain diseased conditions in which the muscular substance is hypertrophied and the motions of the heart strengthened. The paper, which is published in the Medical Times and Gazette for September 12th, 1857, is illustrated by two wood cuts, one of the heart of a child, the other of a portion of that of a heifer. From these it would appear that the cardiac or aortic plexus, long since described by Fallopius, gives off numerous branches, which ramify in all directions over the surface of the heart, swelling here and there in ganglionic enlargements, especially where they cross blood-vessels. These nerves are all soft, flat and somewhat semi-transparent, of a gray color, and invested as to their smaller branches with a neurilemma.

The author states that the chief difficulty in the demonstration of these nerves, arises not from their softness nor the amount of fat in which they are imbedded, but from the presence of a dense fibrous tissue closely investing the heart and sending out prolongations along the vessels and nerves, as well as the fasciculi of muscular fibre. This tissue, which he describes as lying directly under the pericardium, he calls the cardiac fascia.

4.—*Circulation in the Capillaries.*—Dr. Austin Flint publishes a paper on this subject, in the July number of the American Journal of Medical Sciences. Our space will not permit to transcribe the whole article, or to follow him through his very interesting experiments. We must, therefore, content us with a brief statement of the conclusions at which he has arrived.

After passing in review the various theories of the circulation, and condemning Dr. Draper's notion of the predominance of mere capillary attraction, as utterly untenable, and Harvey's theory as too exclusive, he recounts the experiments which have led him to his conclusion. The result of his inquiries is that there is a distinct capillary action, depending chiefly upon the vital attraction between the circulating liquid and the adjacent tissues arising from the processes of nutrition. He admits that the *vis a tergo* of the heart has a great deal to do with the capillary circulation, and does not deny the suction power of the veins, but lays the greatest stress upon the attraction just mentioned, which he calls the capillary power.

The necessary conditions of the healthy performance of the capillary circulation are—

"First, a healthy condition of the vital particles, which is produced by healthy nutrition. Secondly, a particular condition of the blood which is produced by respiration."

With regard to the latter condition he remarks:

"When the process of respiration or æration of the blood is checked, the blood cannot circulate. This we know to be the fact, but we demonstrate that, in asphyxia, the impediment to the circulation takes place in the capillaries; that the condition of oxygenation is necessary to the performance of the vital functions, and it may be that the entire want of the 'capillary power' throws all the onus on the heart, and that the heart is insufficient for the labor. In one of my experiments, after the capillary circulation had entirely ceased, the chest was opened and the heart found beating regularly."

---

5.—*Perineuron, a New Element in Nerves.*—Dr. C. Robin has recently described a new tissue altogether distinct from the neurilemma investing nervous matter. Its characters are—

1. Its situation around the primitive bundles of nerve-tubes and sometimes around the tubes themselves.

2. Its tubular form and its ramifications accompanying the ramifications of the nerves.

3. Its chemical reactions with various tests.

4. A thin, transparent wall, finely granulated, often presenting longitudinal striæ and nuclei, more numerous in the small tubes than in the large.

The perineuron is to the nerve-tubes what the sarcolemma is to the muscular fibres, the immediate investment of the proper nerve-tissue. It is found in all the nerves of animal life, including the vagus, in all their length from the ganglia for the sensitive nerves, and for those which do not pass through ganglia, from the cerebro-spinal axis. The three nerves of special sense are the only exceptions to this, they not having the perineuron. In the sympathetic nerve, this envelop exists only where there are white bundles or ramifications, and not among the gray or gelatiniform bundles.

In large nerves, the tubes of the perineuron are large (two to five-tenths of a millimetre,) in ramifications they become smaller, containing then only two or three nerve-tubes, and sometimes only one. The capillaries do not go inside these tubes.

*Thyroid Gland.*—Dr. Peter Martyn has communicated to the Royal Society some very ingenious speculations as to the use of this remarkable body. He first calls attention to the necessity of rigidity in all round instruments. This is accomplished, he thinks, by the thyroid gland, which, being pressed upon by the muscles, during the act of speaking, becomes gorged with blood, and presses upon the larynx, rendering it tense. Furthermore, he believes this so-called gland acts as a loader. In musical instruments, loaders are used to render the vibrations slower and longer, and the tone in consequence fuller, louder and deeper. They thus give to the voicing part of a small instrument the power aud quality of a large one. The human organ of voice is only three inches long, and yet "has the same power as and better quality of tone than the instrument which more nearly approaches it—the French horn—which is 9 feet, or the 'vox humana' pipe of a moderate sized organ, which is from 4 to 8 feet long. This economy of size in the human organ has always been wondered at, but never, that I know, explained." Finally, the author of the paper believes that by the varying shape, bulk, density and pressure of this body, it aids materially in producing the qualities of modulation and expression belonging to the human voice.

